# Jaundice-Reducing Efficacy Through Placement of Biliary Plastic Stents During Endoscopic Retrograde Cholangiography in Unresectable Malignant Hilar Biliary Obstructions

**DOI:** 10.7759/cureus.87994

**Published:** 2025-07-15

**Authors:** Songming Ding, Aili Lu, Shanjie Dong, Hengkai Zhu, Yiting Hu, Shusen Zheng, Qiyong Li

**Affiliations:** 1 Division of Hepatobiliary and Pancreatic Surgery, Shulan (Hangzhou) Hospital, Zhejiang Shuren University, Hangzhou, CHN; 2 Division of Oncology, First Affiliated Hospital, Zhejiang University School of Medicine, Hangzhou, CHN

**Keywords:** carbohydrate antigen 19-9 (ca19-9), endoscopic retrograde cholangiography, gamma glutamyltranspeptidase (ggt), lanine aminotransferase (alt), unresectable malignant hilar biliary obstruction (umhbo)

## Abstract

Objective: To evaluate the efficacy of endoscopic retrograde cholangiography (ERC)-guided biliary drainage as a preliminary method for reducing jaundice in patients with unresectable malignant hilar biliary obstruction (UMHBO), and to identify risk factors associated with sub-optimal jaundice reduction.

Methods: A cohort of 33 patients with UMHBO, spanning from March 2016 to July 2024, was included in the study. A 30% reduction in total bilirubin (TB) was considered indicative of a favorable jaundice-reducing effect (TB before discharge/TB before ERC).

Results: The rate of good jaundice-reducing effect was 78.8% (26/33) with the use of biliary plastic stents during the initial ERC. Notably, pre-ERC levels of gamma glutamyltranspeptidase (GGT) and alanine aminotransferase (ALT) were lower in the poor effect group compared to the good effect group (260.0 vs. 479.5 U/L, 55.0 vs. 84.5 U/L, respectively, P < 0.05). Carbohydrate antigen 19-9 (CA19-9) was higher in the poor effect group than in the good effect group (7948.6 vs. 542.1 U/ml, P < 0.05). However, binary logistic regression analysis did not reveal that pre-ERC levels of GGT, ALT, and CA19-9 were independent risk factors for poor jaundice reduction.

Conclusion: ERC with the placement of biliary plastic stents as the initial jaundice reducing method was available in UMHBO patients. Independent risk factors that lead to poor jaundice reduction were still elusive.

## Introduction

A malignant hilar biliary obstruction (MHBO) is usually a lethal condition and poses a high challenge to clinical physicians [1]. For most patients suffering from MHBO, the chance of radical surgery has been lost. Their life expectancy is circumscribed, with a five-year survival rate of less than 10%. Besides, they are afflicted with distressing and debilitating symptoms, and intractable pruritus is a particularly troublesome one [2].

Endoscopic retrograde cholangiography (ERC)-guided biliary drainage, commonly abbreviated as ERC-BD, is an efficient medical intervention to improve the quality of life (such as free of pruritis) in patients with unresectable MHBO (UMHBO) [3]. It also prolongs life by decreasing hyperbilirubinemia so as to allow chemotherapy. ERC-BD has been used for several decades. Despite its widespread use and recognized benefits, the optimal endoscopic technique for performing biliary drainage in cases of UMHBO continues to be a subject of debate [4-6]. Various approaches and methods have been proposed and studied, yet consensus on the most effective strategy remains elusive. Moreover, the body of research specifically addressing the factors that contribute to the success of biliary drainage in patients with UMHBO is relatively limited. Understanding these factors is crucial for optimizing patient outcomes, as it can guide clinicians in selecting the most appropriate drainage techniques and in tailoring treatment plans to the individual needs of each patient.

In this retrospective analysis, our primary objective was to evaluate the efficacy of ERC with the insertion of plastic stents as an initial treatment approach for patients diagnosed with UMHBO resulting from three distinct types of malignancies: gallbladder cancer (GC), intra-hepatic cholangiocarcinoma (ICC), and hilar cholangiocarcinoma (HCCA), and to identify any potential risk factors that might contribute to a suboptimal response in terms of reducing jaundice.

## Materials and methods

Study design and population

The study protocol was approved by the Research Ethics Committee, Shulan (Hangzhou) Hospital (Approval No. KY2024134, dated December 25, 2024). The inclusion criteria were initial ERC-BD procedures performed in our hospital using biliary plastic stent implantation, and the patients ≥ 18 years of age. The exclusion criteria included: (1) UMHBO patients concomitant with common bile duct stones or intra-hepatic bile duct stones; (2) having a history of liver surgery; (3) having a history of chemotherapy, transarterial chemoembolization, molecular targeted therapy and immunotherapy; (4) receiving percutaneous transhepatic biliary drainage (PTBD) before or after ERC-BD; (5) receiving endoscopic nasobiliary drainage (ENBD) before plastic stent implantation; (6) cholangitis post-ERC and removal of plastic stent for ENBD during the same hospitalization period; (7) patients who were treated with an artificial liver therapy before or after ERC-BD; (8) and drainage duration < 4 days. The study was conducted in accordance with the Declaration of Helsinki.

The final cohort comprised 33 consecutive UMHBO patients who underwent biliary plastic stent implantation during ERC at Shulan (Hangzhou) Hospital, affiliated with Zhejiang Shuren University, between March 2016 and July 2024. The UMHBO included in this study were all Bismuth type IV [7]. A decrease in total bilirubin (TB) by 30% was defined as a favorable jaundice-reducing effect. This was calculated by dividing the most recent serum TB value measured prior to hospital discharge by the most recent serum TB value obtained before ERC. Consistent with the successful drainage criteria defined in a previous study [8]. Most patients with poor jaundice-reducing effect returned to local hospitals for palliative and supportive treatment. The TB level of patients with good jaundice-reducing effect upon readmission met the above criteria. Drainage duration was defined as the interval between ERC performance and the latest serum TB measurement before patient discharge. During the follow-up period, 31 patients had died.

Evaluation indexes

We analyzed multiple factors: age, gender, whether imaging examination indicating liver cirrhosis, the level of albumin (ALB) (35-55 g/l), alanine aminotransferase (ALT) (9-50U/L), aspartate aminotransferase (AST) (15-40U/L), total bilirubin (TB) (0-21 µmol/L), direct bilirubin (DB) (0-5 µmol/L), indirect bilirubin (IB) (3-14 µmol/L), carbohydrate antigen 19-9 (CA19-9) (≤ 43 U/mL), gamma glutamyltranspeptidase (GGT) (7-45 U/L), alkaline phosphatase (AKP) (40-150 U/L) and prothrombin time (PT) (9.4-12.5 s) before ERCP; sphincterotomy status, unilateral/bilateral drainage as well as the highest level of white blood cells (WBC) (3.5-9.5*109/L), C-reactive protein (CRP) (0-10 mg/L), temperature (°C) post ERCP, and drainage duration on the jaundice-reducing effect in patients with UMHBO. 

Statistical methods

Numerical data were reported as the mean ± SD (or median with interquartile range (IQR)), and categorical variables were reported as percentages. The independent Student's t-test or the Mann-Whitney U-test was used to compare differences in the numerical variables. The likelihood ratio chi-square test was used to evaluate differences in the categorical variables. Binary logistic regression (“Enter” methodology) was used to evaluate risk factors associated with sub-optimal jaundice reduction. A P-value < 0.05 was considered significant. Analyses were performed using IBM SPSS for Windows, version 19.0 (IBM Corp., Armonk, NY, USA).

## Results

Baseline characteristics

The jaundice-reducing effective rate in total patients was 78.8% (26/33), in the GC group was 100% (7/7), in the ICC group was 60% (9/15), and in the HCCA group was 90.9% (10/11). There was no significant difference in age between the poor jaundice-reducing effect group and the good jaundice-reducing effect group (66.2 vs. 69.6 years). The proportion of male individuals was higher in the poor jaundice-reducing effect group than in the good jaundice-reducing effect group (85.7% vs. 61.5%), although no significant difference was found, as shown in Table 1.

**Table 1:** Age and gender distribution in patients undergoing ERC-BD ERC-BD: endoscopic retrograde cholangiography-guided biliary drainage; SD: standard deviation Good jaundice-reducing group: a decrease of total bilirubin at least by 30%; Poor jaundice-reducing group: total bilirubin decreased by less than 30% or increased after ERC-BD

Patients	Number	Male ratio (%)	Age (years, mean ± SD)
Total	33	66.7%	66.9 ± 12.0
Gallbladder cancer	7	42.9%	68.0 ± 9.3
Intrahepatic cholangiocarcinoma	15	80.0%	67.6 ± 12.4
Hilar cholangiocarcinoma	11	63.6%	65.4 ± 13.7
Good jaundice-reducing group	26	61.5%	66.2 ± 12.0
Poor jaundice-reducing group	7	85.7%	69.6 ± 12.3

Predictive factors associated with the jaundice-reducing effect

Significant differences were detected in the pre-ERC levels of GGT, ALT, and CA19-9 between the poor jaundice-reducing group and the good jaundice-reducing group (260.0 vs. 479.5 U/L, 55.0 vs. 84.5 U/L, 7948.6 vs. 542.1 U/mL, respectively, P < 0.05), as shown in Table 2.

**Table 2:** Comparisons of numerical variables between the good jaundice-reducing group and the poor jaundice-reducing group ALB: albumin; ALT: alanine aminotransferase; AST: aspartate aminotransferase; CA19-9: carbohydrate antigen 19-9; GGT: γ-glutamyl transpeptidase; AKP: alkaline phosphatase; PT: prothrombin time; TB: total bilirubin; DB: direct bilirubin; IB: indirect bilirubin; Drainage duration: the interval between ERC-BD performance and the latest serum TB measurement before patient discharge; WBC: white blood cells; CRP: C-reactive protein Good jaundice-reducing group: a decrease of total bilirubin at least by 30%; Poor jaundice-reducing group: total bilirubin decreased by less than 30% or increased after endobiliary drainage Quantitative data with normal distribution were presented as mean ± standard deviation, and were analyzed using the independent Student's t-test; Data with non-normal distribution were presented as median (interquartile range), and were analyzed using the Mann-Whitney U-test *: significant difference

Parameter	Good jaundice-reducing group (N = 26)	Poor jaundice-reducing group (N = 7)	Test statistic	P-value
ALB (g/L)	33.6 ± 4.3	32.3 ± 2.9	t-value = 0.730	0.471
ALT^*^ (U/L)	84.5 (72.0)	55.0 (33.0)	Z-value = -2.026	0.043
AST (U/L)	82.0 (72.5)	48.0 (111.0)	Z-value = -0.991	0.322
CA19-9^*^ (U/mL)	542.1 (8993.8)	7948.6 (8189.3)	Z-value = -2.284	0.022
GGT^*^ (U/L)	479.5 (445.3)	260.0 (270.0)	Z-value = -2.114	0.035
AKP (U/L)	474.1 ± 209.5	305.7 ± 134.5	t-value = 2.005	0.054
PT (s)	12.0 (1.8)	11.3 (5.1)	Z-value = -0.242	0.808
TB (µmol/L)	215.6 ± 86.0	287.6 ± 126.3	t-value = -1.777	0.085
DB (µmol/L)	166.4 ± 68.1	222.0 ± 100.3	t-value = -1.730	0.094
IB (µmol/L)	49.2 ± 27.1	65.6 ± 32.5	t-value = -1.361	0.183
Drainage duration (days)	9.0 (9.0)	10.0 (24.0)	Z-value = -0.133	0.894
WBC (10^9^/L)	11.4 ± 4.3	12.1 ± 4.9	t-value = -0.391	0.698
CRP (mg/L)	77.1 ± 50.7	49.5 ± 28.0	t-value = 1.371	0.180
temperature (°C)	38.2 ± 0.9	38.2 ± 0.6	t-value = -0.103	0.919

However, there was no significant difference in ALB, AST, AKP, TB, DB, IB, PT, temperature, WBC, CRP, or drainage duration between the two groups. Meanwhile, there was no significant difference in the proportion of sphincterotomy, liver cirrhosis, or unilateral drainage between the poor jaundice-reducing effect group and the good jaundice-reducing effect group (42.9% vs. 65.4%, 42.9% vs. 15.4%, 28.6% vs. 34.6%, respectively), as shown in Table 3. 

**Table 3:** Comparisons of categorical variables between the good jaundice-reducing group and the poor jaundice-reducing group Good jaundice-reducing group: a decrease of total bilirubin at least by 30%; Poor jaundice-reducing group: total bilirubin decreased by less than 30% or increased after endobiliary drainage; Hyperamylasemia: amylase > 3 times the upper limit of normal value Likelihood ratio test was used to evaluate differences in the categorical variables

Categorical variable	Good jaundice-reducing group (N = 26)	Poor jaundice-reducing group (N = 7)	Test statistic	P-value
Liver cirrhosis (yes,%)	4 (15.4%)	3 (42.9%)	χ^2^ = 2.220	0.136
Sphincterotomy (yes,%)	17 (65.4%)	3 (42.9%)	χ^2^ = 1.149	0.284
Unilateral drainage (yes,%)	9 (34.6%)	2 (28.6%)	χ^2^ = 0.092	0.761
Hyperamylasemia (yes,%)	6 (23.1%)	2 (28.6%)	χ^2^ = 0.088	0.766

The detailed drainage model is shown in Table 4.

**Table 4:** Details of the drainage technique RBDD: right bile duct drainage; LBDD: left bile duct drainage; (-): no biliary plastic stent placement; F = french; cm = centimeter Patient (1-7): poor jaundice-reducing group; Patient (8-33): good jaundice-reducing group

Patient No.	RBDD	LBDD
1	7F × 13 cm	7F × 13 cm
2	-	7F × 11 cm
3	7F × 9 cm	7F × 11 cm
4	7F × 13 cm	7F × 13 cm
5	7F × 11 cm	-
6	7F × 11 cm	7F × 13 cm
7	7F × 15 cm	7F × 15 cm
8	7F × 9 cm	7F × 9 cm
9	-	8.5F × 9 cm
10	7F × 12 cm	7F × 13 cm
11	7F × 13 cm	7F × 15 cm
12	7F × 12 cm	8.5F × 13cm
13	-	8.5F × 13cm
14	7F × 11 cm	7F × 11 cm
15	7F × 13 cm	7F × 13 cm
16	7F × 11 cm	7F × 11 cm
17	7F × 12 cm	7F × 13 cm
18	7F × 13 cm	7F × 15 cm
19	7F × 13 cm	5F × 7 cm
20	8.5F × 13cm	-
21	7F × 13 cm	7F × 15 cm
22	7F × 12 cm	7F × 12 cm
23	8.5F × 9 cm	8.5F × 11 cm
24	7F × 13 cm	7F × 15 cm
25	8.5F × 9 cm	-
26	7F × 11 cm	7F × 9 cm
27	8.5F × 9 cm	-
28	7F × 13 cm	7F × 13 cm
29	-	8.5F × 9 cm
30	7F × 11 cm	-
31	8.5F × 9 cm	-
32	-	8.5F × 13cm
33	7F × 7 cm	7F × 12 cm

Binary logistic regression analysis did not reveal that the above-mentioned variables, including pre-ERC levels of GGT, ALT, and CA19-9, were independent risk factors for poor jaundice reduction, as shown in Table 5.

**Table 5:** Univariate logistic regression analysis of factors associated with poor jaundice-reducing effect OR: odds ratio; 95% CI: 95% confidence intervals; ERC: endoscopic retrograde cholangiography; PT: prothrombin time; ALB: albumin; ALT: alanine aminotransferase; AST: aspartate aminotransferase; WBC: white blood cells; CRP: C-reactive protein; GGT: γ-glutamyl transpeptidase; AKP: alkaline phosphatase; TB: total bilirubin; DB: direct bilirubin; IB: indirect bilirubin; CA19-9: carbohydrate antigen 19-9 “Enter” methodology was used in the binary logistic regression analysis

Factor	OR (95% CI)	P-value
Age	1.026 (0.951-1.106)	0.509
Male	3.750 (0.391-35.923)	0.252
Liver cirrhosis	4.125 (0.657-25.904)	0.131
Sphincterotomy	0.397 (0.072-2.174)	0.287
Unilateral drainage	1.323 (0.213-8.264)	0.764
PT pre-ERC	1.089 (0.723-1.642)	0.682
ALT pre-ERC	0.976 (0.944-1.008)	0.139
AST pre-ERC	0.995 (0.980-1.011)	0.556
GGT pre-ERC	0.995 (0.990-1.000)	0.074
AKP pre-ERC	0.994 (0.987-1.000)	0.068
ALB pre-ERC	0.922 (0.744-1.143)	0.459
TB pre-ERC	1.008 (0.999-1.017)	0.098
DB pre-ERC	1.010 (0.998-1.022)	0.108
IB pre-ERC	1.020 (0.990-1.051)	0.186
CA19-9 pre-ERC	1.000 (1.000-1.000)	0.050
Highest CRP post-ERC	0.985 (0.963-1.007)	0.185
Highest temperature post-ERC	0.947 (0.348-2.577)	0.916
Highest WBC post-ERC	1.040 (0.859-1.260)	0.688
Drainage duration	1.044 (0.957-1.138)	0.333
Amylase post-ERC	1.000 (0.998-1.002)	0.816

Adverse events

There were no severe complications such as intestinal perforation or massive bleeding caused by the ERC-BD. However, the proportion of hyperamylasemia (> 3 times the upper limit of normal value) was not low, with 23.1% in the good jaundice-reducing group and 28.6% in the poor jaundice-reducing group. This may be due to the fact that pancreatic duct stents were not routinely placed during the ERC-BD procedures at our center. In this study, only three patients had 5F pancreatic duct stents left in place. Luckily, none of them experienced severe pancreatitis.

## Discussion

UMHBO, stemming chiefly from HCCA, ICC, GC, the growth of locally advanced gastric cancer, and metastatic liver cancer, gives rise to a wide range of pathophysiological dysfunctions in the liver, kidney, heart, and immune system [9,10]. ERC-BD stands out as the most frequently employed technique to alleviate the symptoms of obstructive jaundice that result from UMHBO. ERC-BD for UMHBO is performed using a plastic stent (nasobiliary drainage tube) or self-expandable metal stent (SEMS) [11,12]. Plastic stents have been a staple in medical practice for over 30 years, favored for their technical ease of use, cost-effectiveness, and straightforward removal process.

In our investigation, the efficacy rate for reducing jaundice in patients with Bismuth type IV biliary obstruction following the implantation of plastic stents was 78.8%. This figure aligned closely with the results of a retrospective study conducted in 2024, which reported an efficacy rate of 72.0% [13].

GGT, ALT, AST, PT, and TB were used as biomarkers of liver function. GGT has been reported to be sensitive to the extent of malignant obstruction in the lower bile duct [14]. In the present study, it was noted that the levels of pre-ERC γ-GGT were significantly lower in the group with an unfavorable therapeutic outcome as opposed to the group with a favorable outcome. However, binary logistic regression analysis did not show that pre-ERC γ-GGT level was significantly associated with a less effective reduction of jaundice in patients with UMHBO who underwent ERC-BD using plastic stents. A higher level of TB is associated with an increased risk of liver failure. It has been established that blood bilirubin levels surpassing 30 mg/dL represent an independent factor for heightened complications and a lack of efficacy in endoscopic ultrasound (EUS)-guided hepaticogastrostomy [15]. In our study, the levels of pre-ERC TB were slightly higher in the group with a poor outcome compared to the group with a good outcome. The proportion of liver cirrhosis was lower in the group with a good jaundice-reduction than in the group with a poor jaundice-reduction. However, we found that pre-ERC ALT levels were significantly lower in the poor effect group compared to the good effect group. Meanwhile, pre-ERC AST and PT levels were slightly lower in the poor effect group compared to the good effect group, without a significant difference. This complex phenomenon may be related to the long-term burden of malignancies on the liver. Therefore, it could be explained that the CA19-9 values in the group with a poor jaundice-reduction were significantly higher than those in the group with a good jaundice-reduction. However, binary logistic regression analysis did not reveal that pre-ERC levels of ALT and CA19-9 were independent risk factors for poor jaundice reduction, either.

A plethora of studies have assessed the efficacy and safety of bilateral as opposed to unilateral biliary drainage in patients with UMHBO [16,17]. Despite this, the decision between unilateral and bilateral drainage remains a subject of debate. A recent systematic review demonstrated that, in the context of UMHBO, bilateral biliary drainage was linked to a lower rate of re-intervention when contrasted with unilateral drainage. Nonetheless, no significant variations were found in terms of technical success or the occurrence of either early-stage or late-stage complications [2]. However, Xia Mingxing from Shanghai Eastern Hepatobiliary Hospital posited that the insertion of bilateral stents was preferable to a single stent in terms of clinical success, stent patency, and overall survival, as evidenced by a substantial multicenter parallel study involving 1,239 patients [18]. However, in this study, we did not determine that unilateral drainage posed a risk factor for a diminished jaundice-reducing effect. It is imperative to consider whether the sample size is inadequate to substantiate the claim that unilateral drainage is a risk factor for a poor jaundice-reducing effect. This matter warrants further investigation.

Cholangitis is frequently encountered in patients with malignancies. Notably, approximately 50% of patients presenting with biliary obstruction exhibit a positive bile culture [19]. A positive bile culture can also occur after manipulation of the biliary tree with concomitant direct or enteric contamination [20]. An episode of cholangitis not only leads to early stent occlusion and aggravates the deteriorating liver function but also triggers multiple organ dysfunction, all of which can have a negative impact on ERC-BD. As a result, it is imperative that all patients undergoing biliary drainage receive a prophylactic antibiotic prior to the ERC-BD procedure, with the antibiotic regimen continued in the event that cholangitis develops following the intervention [21]. Within the framework of our study, we excluded patients who developed acute cholangitis after stent placement to minimize interference with the study. However, cases of cholangitis post-ERC-BD were not uncommon.

Frequently, PTBD was necessitated following insufficient ERC drainage in patients with UMHBO. Numerous reports in the literature have indicated that, for patients diagnosed with potentially resectable perihilar cholangiocarcinoma, the initiation of PTBD as opposed to ERC-BD could potentially lead to more favorable clinical outcomes [22]. Furthermore, for patients with Bismuth III/IV-type tumors, PTBD has been demonstrated to be safer, with lower rates of biliary infection [23]. More recently, EUS-guided biliary drainage has been reported as a promising alternative to ERCP-BD [24]. Additionally, combined ERCP and EUS procedures have been proposed as a means to achieve better drainage in complex cases of MHBO [25].

This single-center retrospective study had several limitations. First, the sample enrolled was relatively small. Second, we did not identify any independent risk factors related to poor jaundice-reduction effect. Third, we did not include cases of ERC-BD combined with PTBD. Because when PTBD and stent drainage were both located on the same side, we could not determine which method had exactly exerted the effect of reducing jaundice. At our center, we preferred to use PTBD or percutaneous transhepatic gallbladder drainage to alleviate acute cholangitis after ERC-BD.

## Conclusions

ERC with biliary plastic stent placement is an effective method for alleviating obstructions in patients suffering from UMHBO. Independent risk factors related to poor jaundice reduction need further exploration. Cholangitis post-ERCP should be controlled.
